# The Role of Food Matrices Supplemented with Milk Fat Globule Membrane in the Bioaccessibility of Lipid Components and Adaptation of Cellular Lipid Metabolism of Caco-2 Cells

**DOI:** 10.3390/nu16162798

**Published:** 2024-08-22

**Authors:** Victoria Martínez-Sánchez, María Visitación Calvo, Javier Fontecha, Antonio Pérez-Gálvez

**Affiliations:** 1 Group of Chemistry and Biochemistry of Pigments, Instituto de la Grasa (CSIC), Building 46, 41013 Sevilla, Spain; v.martinez.sanchez@csic.es; 2 Food Lipid Biomarkers and Health Group, Institute of Food Science Research (CSIC-UAM), 28049 Madrid, Spain; mv.calvo@csic.es (M.V.C.); j.fontecha@csic.es (J.F.)

**Keywords:** bioaccessibility, food matrix, in vitro digestion, intestinal cell model, milk fat globule membrane

## Abstract

This study aimed to evaluate the digestive efficiency of food matrices supplemented with milk fat globule membrane isolated from buttermilk (BM-MFGM), using the INFOGEST in vitro digestion protocol hyphenated with the assessment of the digested material on the lipid profile of the Caco-2 cell culture model. First, we examined lipid profiles in food matrices supplemented with BM-MFGM and their subsequent digestion. The results showed distinct lipid profiles in different food matrices and micellar fractions. The presence of BM-MFGM lipids changed the cellular lipid profiles in Caco-2 cell cultures, with diverging contents in cholesteryl esters, triacylglycerides, and neutral lipids depending on the micellar food matrix factor. Hierarchical clustering analysis revealed patterns in cellular lipid responses to micellar stimuli, while volcano plots highlighted significant changes in cellular lipid profiles post-treatment. Thus, this study underscores the importance of in vitro digestion protocols in guiding food matrix selection for bioactive ingredient supplementation, elucidating intestinal epithelium responses to digested food stimuli.

## 1. Introduction

The rising public awareness regarding the relationship between human health and dietary intake is undeniable. It is common for consumers to look carefully at the labels of food products, whether it is the list of ingredients or the claims that appear on the front. Most of these claims are related to compounds included in the final food product, such as polyunsaturated fatty acids, minerals, or vitamins, frequently linked with beneficial health outcomes. Given their relevance to the population, it is not surprising that the food industry pursues the development of products containing beneficial compounds for human health [1]. However, despite the benefits described, the effects of human digestion on the availability of those compounds are scarcely considered when determining their impact on human health. Nutrients and bioactive compounds with a lipophilic nature must be released from the food matrix and later included in digestive structures that facilitate their absorption by the intestinal system, the micelles. The proportion of nutrients/bioactive compounds released from the food matrix after digestion and their availability for absorption by the cells of the intestinal epithelium, in addition to the percentage that can enter the cells, is defined as bioaccessibility. Nevertheless, this parameter is not invariable, as the nature of the food matrix affects the efficiency of the bioaccessibility, which has been described in numerous studies [2,3,4].

The bioaccessibility of a specific food ingredient, for which interest has increased exponentially in recent years, is starting to be studied in addition to its effect on the lipid metabolism of an intestinal cell model. Intestinal cells are able to sense and modify their lipid metabolism depending on the abundance of polar/non-polar lipid classes assimilated by micelles according to the availability of that specific compound. It is the milk fat globule membrane (MFGM). MFGM contains a distinct mix of complex proteins and lipids (60% and 40%, respectively) derived by mammalian secretory cells during milk production. The lipid fraction mainly consists of phospholipids (PL; around 30% of the total lipid weight of the MFGM) and sphingomyelin (SM) or cholesterol, among other lipid constituents [5]. These constituents have been described to improve the neurobehavioral development of premature infants [6] by inducing the early myelination associated with improved neurodevelopment and cognitive development in the infant population [7]. MFGM modulates the cytokine profile to levels comparable to those of a breastfed infant, supporting the normal functioning of the immune system [8]. Lactadherin, among other MFGM proteins, decreases bloody diarrhea and diarrhea episodes in children [9], and the presence of sphingolipids in MFGM composition shapes the microbial population in the early stages of life [10]. Moreover, it also benefits cardiometabolic health by attenuating adipose tissue inflammation and hepatic steatosis [11], decreasing hepatic accumulation of cholesterol [12], and decreasing cholesterol absorption in humans [13], making MFGM an interesting ingredient to be supplemented into food products. Buttermilk (BM), a traditional food-grade by-product from the dairy industry, has been employed as a source of MFGM due to its high concentration of polar lipids [14,15,16]. A concentrate of MFGM from BM (BM-MFGM) was prepared to study the possibilities of its use as a future food supplement to slow progression of age-related cognitive decline [17], although the use of in vitro models comes first to study the molecular mechanisms and their effect on the intestinal cell lipid metabolism after an in vitro digestion protocol.

While evidence regarding the benefits of MFGM as a food ingredient is growing, additional efforts should be focused on determining the appropriate food matrix or matrices that provide consumers with the benefits of the MFGM. In vitro digestion protocols have emerged as a significant methodology to provide with data of the digestive fate and digestibility efficiency of food components, revealing the main effectors either enhancing or decreasing that efficiency, and yielding insights for a fine processing and product formulation. However, in vitro digestion data are not the endpoint but require inputs from experimental cell culture models aimed to measure the cellular absorption and trafficking of digested material. Indeed, it is the combination of digestion protocols with cell culture methods in a high-throughput fashion that yields in-depth working conclusions from data evaluation. Such conclusions would drive attention not only to efficient digestion but also to the metabolic changes in the cellular tissue. The aim of this study was to determine the digestive efficiency of BM-MFGM supplemented in food matrices with different composition through a standardized in vitro digestion protocol and to assess the impact of the digested material on the lipid profile of the cell culture. Supervised statistical techniques were applied to deal with the high amount of lipid features characterized in the micelles and in the cells. Intra- and inter-comparisons were made to unravel the lipid features, which are descriptors for the digestion and/or cellular metabolism, depending on the food matrix factor as the main effector. Thus, the selection of the appropriate food matrix and the prediction of adaptative processes of the cell culture could be anticipated.

## 2. Materials and Methods

### 2.1. Preparation of BM-MFGM

BM-MFGM concentrate was isolated as described previously [17], using a pilot-scale filtering system in an ultrafiltration-diafiltration (UF-DF) process at the Innolact (Lugo, Spain) facilities from a liquid pasteurized BM, provided by a dairy producer. After the initial UF phase, the permeate from the BM was removed, leaving behind a concentrated BM. To offset the rise in viscosity and lower the lactose concentration in the retentate, water was then cycled back through the system. The mixture was then ultrafiltered, and after removing the permeate, BM that was eight times as concentrated (BM-MFGM) as the starting material was obtained. BM-MFGM was evenly distributed in a layer, frozen at −80 °C, and then lyophilized in a Lyobeta-15 Freeze-Dryer (Telstar Technologies, Terrassa, Spain). The lipid composition of BM-MFGM is detailed in Table S1.

### 2.2. Experimental Design

To study the effects of the matrix composition on the bioaccessibility of BM-MFGM, three types of food formulations were prepared following the protocols originated in our laboratory: jelly (protein matrix, JM), cookie (carbohydrate matrix, CM), and cookie with sunflower oil (lipid- and carbohydrate-matrix, LCM). Briefly, after a hydration step of the BM-MFGM to facilitate the dissolution of the compound (18 h at 4 °C), three levels of supplementation of BM-MFGM were studied in each food formulation (2%, 5%, and 10% *w*/*w*). The weight of BM-MFGM necessary to reach each level of supplementation was added and dissolved directly in the food matrix. The ingredient composition of each food matrix is described in Table 1. For the JM, all the ingredients were mixed and heated until complete dissolution and cooled until complete solidification of the matrix (4 °C). For the CM and LCM, the ingredients were mixed and baked at 180 °C for 15 min. Each preparation was kept frozen (−20 °C) until needed. Each food matrix supplemented with BM-MFGM (2%, 5%, and 10%) and controls (0%), followed the static INFOGEST in vitro digestion protocol.

### 2.3. In Vitro Digestion

The standard static INFOGEST digestion protocol [18], with the modifications described previously [19] for each food matrix, was followed in this study. These modifications were required to isolate the micellar fraction (MF) of the food matrices, which were filtered and aliquoted for lipid extraction of the micelles and for performing the cell experiments. In contrast, lipid fraction was extracted from micelles and cellular cultures afterwards for further characterization. As described, simulated digestion fluids were prepared in advance and frozen until needed (−20 °C). Preliminary experiments were performed to adjust the pH to the gastric and intestinal phases of each food matrix and each BM-MFGM supplementation. On the day of the experiment, solutions and enzymes were thawed and prepared before use. Three replicates of each sample in three different digestion procedures were followed.

### 2.4. Caco-2 Cell Culture, Protein Quantification, and Cell Viability

Maintenance of the cell culture of Caco-2 was performed by culturing the cells in 75 cm^2^ flasks in low glucose DMEM medium with L-glutamine and sodium pyruvate (Gibco, Fisher Scientifics, Waltham, MA, USA), supplemented with 10% heat-inactivated fetal bovine serum (FBS, Fisher Scientifics, Waltham, MA, USA), 1% penicillin/streptomycin (Gibco), and 1% non-essential amino acids (Gibco) (complete medium) under a 37 °C and 5% CO_2_ atmosphere. After reaching 70–90% of confluency, weekly subculture was performed using 0.25% trypsin-EDTA (Gibco) and seeded on 96-well plates (Corning, Fisher Scientifics, Waltham, MA, USA) for cell viability and 6-well plates (Corning) for MF incubation experiment at a density of 2.1 × 10^4^ per cm^2^. The cell culture was fully differentiated after 21 days, with culture media changed every other day. To obtain three digestion replicates, filtered MF replicates from the same concentration and digestion were pooled together. The day before the experiment, complete medium was replaced to FBS-free medium. On the day of the experiment, MF samples were diluted to avoid cell cytotoxicity (previously tested) according to the following steps: MF samples from JM and LCM were diluted, firstly, 1:4 in Hank’s Balance Salt Solution (HBSS, Gibco) and, subsequently, 1:3 in FBS-free medium, while MF samples from CM were diluted 1:2 in HBSS and, later, 1:3 in FBS-free medium and incubated with Caco-2 cells at 37 °C for 4 h and 5% CO_2_ atmosphere. Three replicates of control cells and levels of BM-MFGM supplementation (0, 2, 5, and 10%) were used in each experiment. After incubation, cells were washed with ice-cold 10 mM sodium taurocholate and the monolayer was scraped with 1 mL of 10% ethanol buffer saline four times and placed in 50 mL Falcon tube. Samples were kept frozen (−20 °C) until use. In order to normalize the data, protein concentration was determined by BCA protein assay (Novagen, Merck KGaA, Darmstadt, Germany). To determine cell cytotoxicity of MF samples, Neutral Red (NR) assay was performed [20,21]. Briefly, MF samples were diluted, and after 4 h of incubation with Caco-2, the cells were washed with HBSS. 50 µg/mL of NR in a complete medium was added and incubated for 30 min at 37 °C under a 5% CO_2_ atmosphere. After incubation, the solution was discarded. Cells were lysated with 50% ethanol buffer saline containing 1% acetic acid, and the NR inside of the viable cells was released. The absorbance of the released NR was measured at 570 nm using Multiskan Spectrum spectrophotometer (Thermo, Fisher Scientifics, Waltham, MA, USA) at room temperature (25 °C).

### 2.5. Isolation Protocol of Lipids from Food Matrices, Micellar Fractions, and Cell Culture

The extraction of the lipid fraction from food matrices, MF samples, and Caco-2 cell culture was performed following a simplified method [15], but with some modifications detailed as follows. At 25 °C, one sample volume was mixed with one volume of methanol and vortexed at maximum speed (2000 rpm) (Multi Reax; Heidolph Instruments GmbH & Co. KG, Schwabach, Germany) for 10 min. Later, two additional volumes of dichloromethane were added followed by two rounds of 10 min vortex at maximum speed, intercalating 1 min of ultrasonication. A final 0.4 volume of water with 1% acetic acid was included and vortex at maximum speed mixing applied for 10 min. A final centrifugation was performed (400× *g*, 20 min), and the organic phase (bottom layer) was extracted. N_2_ gas was flushed to evaporate the organic phase. The remaining lipids were weighed and frozen (−20 °C) until chromatographic analysis.

### 2.6. Lipids Analyses

Analysis of triacylglycerides (TAG) and cholesterol was made by GC-FID using a Clarus 400 GC (Perkin Elmer Ltd., Beaconsfield, UK) equipped with an automatic split/splitless injector and a flame ionization detector. A fused-silica capillary column, Rtx-65TAG (30 m × 0.25 mm i.d. × 0.1-μm film thickness; Restek Corp., Bellefonte, PA, USA), was utilized. The chromatographic conditions were set with the following temperature program: an initial hold at 120 °C for 30 s, followed by a ramp of 10 °C/min up to 220 °C with a hold for 30 s, and then a further increase at 6 °C/min up to 350 °C, which was maintained for 30 min. The injector and flame ionization detector were set at temperatures of 355 °C and 370 °C, respectively. Helium served as the carrier gas at 172 kPa, and the injection volume was 0.5 μL of a fat dilution (30 mg/mL) in hexane. The methodology has been described by Castro-Gómez et al. [22]. Lipid compounds determined by this technique are detailed in Table S2. The fatty acids (FA) composition was determined following the chromatographic conditions described by [23]. The GC-FID analysis was performed using an Autosystem chromatograph (Perkin Elmer, Beaconsfield, UK) equipped with a VF-23 ms fused-silica capillary column (30 m × 0.25 mm i.d. × 0.25 μm film thickness, Varian, Middelburg, The Netherlands) and a flame ionization detector (FID). The column temperature was initially held at 60 °C for 1 min after injection, then increased at a rate of 10 °C/min to 130 °C, followed by a slower increase of 3 °C/min to 170 °C, and finally ramped up at 10 °C/min to 230 °C, where it was maintained for 5 min. Helium served as the carrier gas, with the column inlet pressure set to 20 psig and a split ratio of 1:20. The injection volume was 0.5 μL, and the total run time was 32 min. The injector and detector temperatures were maintained at 250 °C and 270 °C, respectively. For the determination and quantification of FAMEs, anhydrous milk fat (reference material BCR-164) from Fedelco Inc. (Madrid, Spain) and tritridecanoine as an internal standard (Sigma, St. Louis, MO, USA) were used. The FA and the grouped families according to unsaturation level and chain length are detailed in Table S3. Analysis of the lipid classes profile was accomplished by HPLC (model 1260; Agilent Technologies Inc., Palo Alto, CA, USA) coupled with an Evaporative Light-Scattering Detector (SEDEX 85 model; Sedere SAS, Alfortville, Cedex, France); the system utilized prefiltered compressed air as the nebulizing gas at a pressure of 350 kPa and a temperature of 60 °C, with the gain set to 3. Two columns in series (250 × 4.5 mm Zorbax Rx-SIL columns with 5-μm particle diameter; Agilent Technologies Inc., Palo Alto, CA, USA) and a pre-column with identical packing material were employed. Prior to analysis, samples were dissolved in CH_2_Cl_2_ at concentrations of either 5 or 30 mg/mL, and 50 μL was injected following column equilibration at 40 °C. The solvent gradient was applied as detailed by Castro-Gómez et al. [22]. The methodology has been described by [15]. Lipid classes and the groups among them are shown in Table S4.

### 2.7. Statistical Analysis

Data from lipids profile features, MF, and cell cultures were analyzed in the model with ‘Food matrix’, ‘Micelles class’ and ‘Cell class’ factor, respectively, and normalized by the median and Pareto scaled. Multivariate analysis assessed data quality by comparing individual samples’ data against the pooled samples. First, using supervised analyses such as partial least squares discriminant analysis (PLS-DA), the different variances were aggregated, denoting significant lipid features according to *p* < 0.05 and variable importance in projection (VIP > 1) scores. Comparing the lipid profiles in MF with those in cell cultures, normalized data of paired samples were subjected to hierarchical cluster analysis using the Ward algorithm and measuring Euclidean distance. These data were also subjected to ANOVA analysis. Raw-*p* values were adjusted for hypothesis testing at a false discovery rate of 5% (FDR ≤ 0.05). A comparison of lipid features from the treated cell cultures with control cells first, and then with cells treated with control food matrices, was performed with the same approach but unpairing the data. The IBM SPSS Statistics for Windows, version 28 (IBM Corp., Armonk, NY, USA) was used for data analysis.

## 3. Results

### 3.1. Digestibility of BM-MFGM Lipids

**Figure 1:**
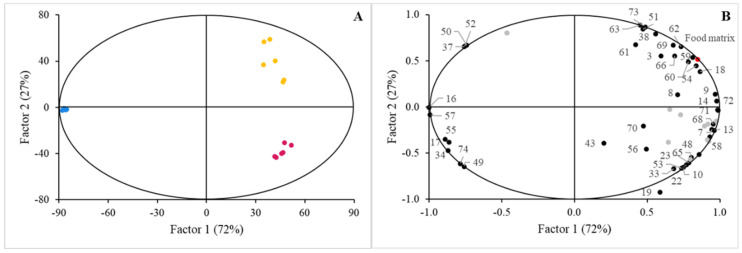
PLS−DA scores plot (**A**) and correlation loadings (**B**) for lipid profiles in LCM (blue circles), JM (red circles), and CM (yellow circles) supplemented with BM−MFGM considering the ‘Food matrix’ as the main factor. The complete list of lipids determined in the study is detailed in Tables S2–S4. Significant lipid features are identified with numbers: 3, triacylglyceride species carbon atom number (CN) CN28; 7, CN36; 8, CN38; 9, CN40; 10, CN42; 13, CN48; 14, CN50; 16, CN54; 17, Ʃneutral species; 18, Ʃpolar species; 19, cholesterol; 20, C10:0; 21, C10:1; 22, C12:0; 23, C14:0; 33, C18:0; 34, C18:1 c9; 37, C18:2; 38, C18:3; 43, C20:4 ω6 (AA); 48, Ʃsaturated fatty acids; 49, Ʃmonounsaturated fatty acids; 50, Ʃpolyunsaturated fatty acids; 51, ω3 fatty acids; 52, ω6 fatty acids; 53, Ʃshort chain fatty acids; 54, Ʃmedium chain fatty acids; 55, Ʃlong chain fatty acids; 56, cholesteryl esters; 57, triacylglycerides; 58, diacylglycerides; 59, free fatty acids plus cholesterol; 60, monoacylglycerides; 61, glucosylceramides; 62, gangliosides; 63, lactosylceramides; 65, phosphatidylethanolamine; 66, phosphatidylinositol; 68, phosphatidylcholine; 69, sphingomyelin; 70, lyso-phospholipids; 71, Ʃpolar lipids; 72, total polar lipids; 73, Ʃceramides; 74, Ʃneutral lipids. Performance of the model is denoted in Table S5.

The performance of the model analysis in terms of accuracy, total explained variance (R^2^), and predictable variation (Q^2^) are described in Table S5, while Figure 1 depicts the scores and correlation loadings plots. The features of the discrimination were noted by the analysis of significant values for the variable importance in projection (VIP) scores (VIP > 1) represented in Table 2.

Despite the consistent use of the BM-MFGM for supplementation across the selected food matrices, a comparative analysis of the lipid classes profile in the final food products exhibited the discriminatory capabilities of several lipid classes when the factor ‘Food matrix’ was selected in the PLS-DA test. As expected, LCM samples clearly correlate with lipid classes featuring the oily nature of this product, such as TAG, neutral lipids, and fatty acids with different chain lengths. Therefore, it seems that the presence of sunflower oil in the food formulation introduced a discriminant effect correlated with the non-polar nature of the lipid classes, but that effect is not related to the composition of the BM-MFGM added to the formulations.

**Figure 2:**
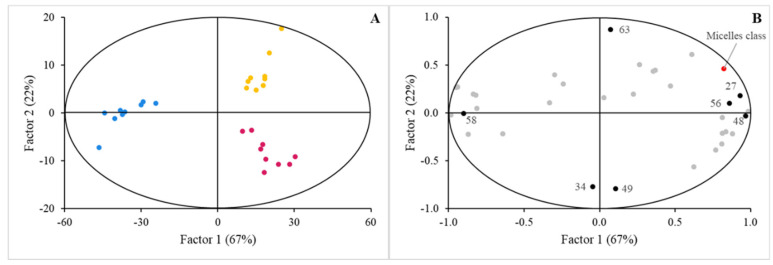
PLS−DA scores plot (**A**) and correlation loadings (**B**) for lipid profiles in micellar fractions isolated from digested food matrices (LCM (blue circles), JM (red circles), and CM (yellow circles), respectively, supplemented with BM−MFGM considering the ‘Micelles class’ factor. The complete list of lipids determined in the study is detailed in Tables S2–S4. Significant lipid features are identified with numbers: 27, C16:0; 34, C18:1 c9; 48, Ʃsaturated fatty acids; 49, Ʃmonounsaturated fatty acids; 56, cholesteryl esters; 58, diacylglycerides; 63, lactosylceramides. Performance of the model is denoted in Table S5.

In a similar fashion, once the BM-MFGM supplemented matrices were digested, the profile of the lipids classes that reached micellarization depending on the factor ‘Micelles class’ was analyzed. Those from JM and CM were located on the same side of the map scores plot, while the MF samples from digested LCM were clustered on the other side of that plot. Markedly, these were fine predictors of the discriminant factor that features the scores plot for the MF samples obtained from digested LCM, raised from the digestion of the original TAG, along with other non-polar lipid classes reaching the highest VIP scores. On the other hand, MF obtained from digestion of the JM or CM was significantly linked to polar lipids, that is, some of the lipid classes characterizing the BM-MFGM. The impact of this factor on the final MF lipid profiles was anticipated, consistent with the observations in Figure 1. The discrimination effect of the factor ‘Micelles class’ is represented in Figure 2. The performance of the corresponding PLS-DA model is represented in Table S5. This behavior was associated with the correlation loadings plot (Figure 2) and the VIP scores represented in Table 2.

### 3.2. Sensing of Cell Cultures to Lipid Micellar Contents

**Figure 3:**
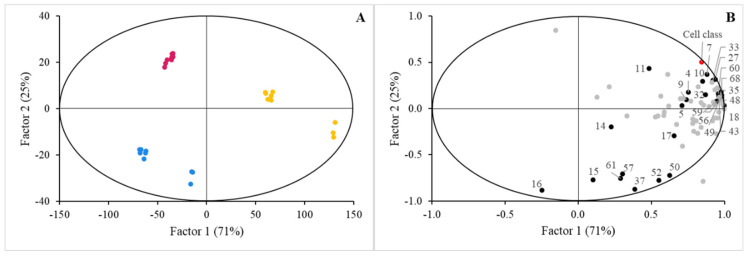
PLS−DA scores plot (**A**) and correlation loadings (**B**) for lipid profiles in cell cultures supplemented with micellar fractions isolated from digested food matrices (LCM (blue circles), JM (red circles), and CM (yellow circles), respectively) supplemented with BM−MFGM considering the ‘Micelles class’ factor. The complete list of lipids determined in the study is detailed in Tables S2–S4. Significant lipid features are identified with numbers: 1, triacylglyceride species carbon atom number (CN); 4, CN30; 5, CN32; 7, CN36; 9, CN40; 10, CN42; 11, CN44; 14, CN50, 15, CN52, 16, CN54; 17, Ʃneutral species; 18, Ʃpolar species; 27, C16:0; 32, C17:1 c10; 33, C18:0; 35, C18:1 c11; 37, C18:2; 43, C20:4 ω6 (AA); 48, Ʃsaturated fatty acids; 49, Ʃmonounsaturated fatty acids; 50, Ʃpolyunsaturated fatty acids; 52, ω6 fatty acids; 56, cholesteryl esters; 57, triacylglycerides; 59, free fatty acids plus cholesterol; 60, monoacylglycerides; 61, glucosylceramides; 68, phosphatidylcholine. Performance of the model is denoted in Table S5.

Next, we questioned if the cells could sense the different MF compositions, that is, considering the ‘Cell class’ as the main effector, and what kind of changes in cellular lipid profiles were produced after the incubation time. Figure 3 depicts the PLS-DA model that describes the significant separation of the cellular lipid profiles depending on the origin of the MF that was applied for incubation. The performance of the model is shown in Table S5, while the VIP scores of the cellular lipid classes are included in Table 2. Incubation of Caco-2 cells MF enriched in compounds derived from the hydrolysis of TAG caused a metabolic shift within the cells towards the accumulation of the precursor’s components for TAG synthesis. Therefore, Caco-2 cells could sense the MF samples originated from the selected food matrices where the BM-MFGM was incorporated. In this case, the feature that first discriminated through the first component axis was the cell cultures incubated with MF originating from the CM. In addition, the spatial distribution of data from the cells incubated with the MF originating from LCM and JM did not cluster sufficiently, as noted in the results presented so far. Thus, it was possible to discriminate between the data at the 2% BM-MFGM supplementation level and those at the 5% and 10% levels that clustered together. This was the single occasion in this study where data were discriminated according to supplementation percentage, pointing to a presumed saturation effect of BM-MFGM.

**Figure 4:**
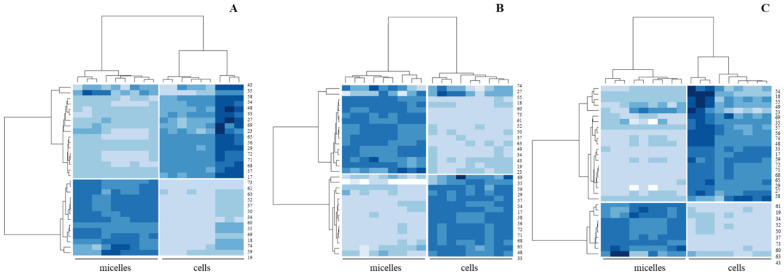
Heatmaps generated through hierarchical cluster analysis for paired comparisons of lipid profiles from micelles, isolated from digested food matrices (LCM, (**A**); JM, (**B**); CM, (**C**)) supplemented with BM-MFGM, and from the cell cultures treated with each micelle class. Significant lipid features are identified with numbers: 17, Ʃneutral species; 18, Ʃpolar species; 19, cholesterol; 23, C14:0; 27, C16:0; 29, C16:1 c9; 33, C18:0; 34, C18:1 c9; 35, C18:1 c11; 37, C18:2; 43, C20:4 ω6 (AA); 48, Ʃsaturated fatty acids; 49, Ʃmonounsaturated fatty acids; 50, Ʃpolyunsaturated fatty acids; 52, ω6 fatty acids; 54, Ʃmedium chain fatty acids; 55, Ʃlong chain fatty acids; 56, cholesteryl esters; 57, triacylglycerides; 58, diacylglycerides; 59, free fatty acids plus cholesterol; 60, monoacylglycerides; 61, glucosylceramides; 63, lactosylceramides; 65, phosphatidylethanolamine; 68, phosphatidylcholine; 69, sphingomyelin; 71, Ʃpolar lipids; 72, total polar lipids; 73, Ʃceramides; 74, Ʃneutral lipids.

A second statistical analysis of the data of the cellular lipid profile was performed and drawn with that of the MF applied in the cell cultures. This was accomplished by comparing the micellar and cellular lipid profiles through hierarchical clustering analysis. The resulting heatmaps are represented in Figure 4.

**Figure 5:**
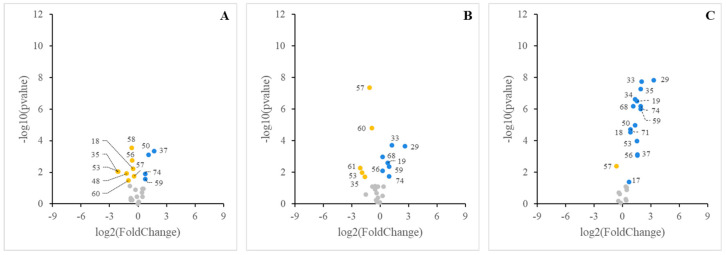
Volcano plots showing changes in lipid features analyzed in cell cultures treated with micelles isolated from digested food matrices (LCM, (**A**); JM, (**B**); CM, (**C**)) supplemented with BM−MFGM. Lipid features with significance of fold change in experimental vs. control cells are indicated for downregulated (yellow circles) and upregulated (blue circles) lipid features. No significant fold change lipid classes are included (grey circles). Lipid features are identified with numbers: 17, ƩNeutral species; 18, Ʃpolar species; 19, cholesterol; 29, C16:1 c9; 33, C18:0; 34, C18:1 c9; 35, C18:1 c11; 37, C18:2; 48, Ʃsaturated fatty acids; 50, Ʃpolyunsaturated fatty acids; 53, Ʃshort chain fatty acids; 56, cholesteryl esters; 57, triacylglycerides; 58, diacylglycerides; 59, free fatty acids plus cholesterol; 60, monoacylglycerides; 61, glucosylceramides; 68, phosphatidylcholine; 71, Ʃpolar lipids; 74, Ʃneutral lipids. Lipid features with significance of fold change in experimental vs. cells.

The contrast of the lipid profile of cell cultures treated with each micellar fraction vs. the control cell culture is represented as volcano plots in Figure 5. This representation easily allowed to note down the lipid features that significantly decreased or increased their content, as well as those that were not affected by the micellar sensing. Additionally, the comparison of lipid features that significantly changed in the experimental vs. the cells treated with food matrices not supplemented with BM-MFGM was performed. In this case, the lipid features that changed significantly were the Ʃneutral lipids that increased in the lipids profile of the cells treated with any of the supplemented BM-MFGM food matrices, reaching 1.69-, 1.90-, and 3.81-fold changes for supplemented LCM, JM, and CM, respectively. Phosphatidylcholine also increased significantly in the lipid profile of the cells treated with BM-MFGM JM or CM, reaching 1.20- and 2.23-fold changes for supplemented JM and CM, respectively. Cholesteryl esters also increased significantly in the cells incubated with supplemented BM-MFGM JM or CM, reaching 1.18- and 2.93-fold changes for supplemented JM and CM, respectively. Finally, triacylglycerides, which significantly decreased in the cells treated with BM-MFGM LCM, JM, or CM; reached 0.75-, 0.46-, and 0.64-fold decreases for LCM, JM, and CM, respectively.

## 4. Discussion

The potential applications of in vitro digestion protocols go beyond quantifying the efficiency of bioaccessibility and/or classifying any significant ‘cause and effect’ on it. Indeed, their nexus with intestinal cell culture models allows the visualization of the inherent ability of the gut epithelium to functionally adapt to the *stimuli* that digested food triggers in the culture. Definitively, the visualization of such effects is more enriching when the changes during both the in vitro digestion and the assimilation of a complex mixture of target bioactive compounds are followed. This conceptual idea was applied in the experimental design of this study and for both data analysis and results presentation.

Initially, our results highlight the impact of food matrix on the bioaccessibility of lipids. Calvo-Lerma et al. [24] reported that the physical and chemical organization between lipids and components of food matrices significantly influences the lipolysis during digestion and, therefore, the release and later absorption of fatty acids in the gastrointestinal tract. Similarly, McClements et al. [25] discussed the critical role of food matrices in the bioavailability of lipophilic compounds like carotenoids, vitamins, flavonoids, and ω3-fatty acids which present low and variable bioavailability due to the poor liberation from food matrices and low solubility in the gastrointestinal fluids which increases the difficulty to be absorbed in the gastrointestinal tract. These hurdles caused by the food matrix reinforced the importance in the selection of the food matrix as it is crucial in functional food development and the need for the careful design of food products to enhance the delivery of bioactive compounds. Similarly, Berry et al. [26] determined that the nature of the food matrix is essential in the development of dietary strategies to modulate metabolic lipid disorders due to the intrinsic relation of the lipid bioaccessibility with the composition of the food matrix. In our study, we observed that lipid- and carbohydrate-rich matrices (LCM) significantly promoted the micellarization of non-polar lipids raised from the digestion of the original TAG along with other non-polar lipid classes reaching the highest VIP scores. In contrast, protein-rich matrices (JM) and carbohydrate-based matrices (CM) favored the micellarization of polar lipids, aligning with the intrinsic lipid composition of BM-MFGM (Figure 2, Table 2). Therefore, the food matrix exerts a significant role in promoting the micellarization of specific lipids. This effect presents important implications for the design of functional foods, as incorporating BM-MFGM into food matrices that favor the micellarization of polar lipids could enhance the delivery of these bioactive components to the intestinal epithelium, maximizing their health benefits [27,28,29,30]. As an example of how components of food matrices can enhance the bioavailability of beneficial compounds for the human health, McClements and Xiao [31] described that lipophilic nutraceuticals, such as carotenoids or phytosterols from vegetables sources, present in dressing or sauces are formulated with vegetable oils, which helps to extract the hydrophobic biomolecules from the plant tissue, increasing their absorbance across the gastrointestinal tract.

Secondly, our study highlights the adaptive capacity of Caco-2 cells in response to varying lipid profiles from the different micellar fractions as observed by the changes in cholesterol esterification and TAG synthesis, reflecting the dynamic nature of the intestinal response to dietary stimuli. This adaptability is essential for maintaining lipid homeostasis, extensively discussed in the lipid absorption and metabolism context [32]. Reboul [33] thoroughly discussed the regulation of fat-soluble micronutrient absorption by the intestinal cells including the role of cellular lipid metabolism in adapting to dietary changes. However, this lipid homeostasis has been observed to be altered in short-term, high-fat diets where the enterocyte cells modify the expression of intestinal genes involved in lipoprotein metabolism as plasma cholesterol, LDL cholesterol, and HDL cholesterol concentrations increases after the three days of a high-fat diet, along with the expression of intestinal genes related with fatty acid metabolism and lipoprotein metabolism such as SREBP-2 (sterol regulatory element binding protein 2), PPAR-α (peroxisome proliferator-activated receptor α), FABP2 (fatty acid binding protein 2), or ACAC-α (acetyl-CoA carboxylase α) [34]. The type and amount of dietary fat can modulate the expression of genes involved in lipid metabolism in liver and adipose tissue, similarly to the intestinal cells, as high-fat diets rich in saturated fats reduce the expression of genes related to fatty acid oxidation, while the consumption of food rich in PUFA can reverse the negative effects of the high-fat diet [35]. However, our results suggest that it is necessary to consider almost the complete cellular lipid profile to explain the underlying phenomena of the cell sensing to MF of different origin, so that each digested food matrix produced a response of the cell culture in terms of lipid profile characteristics because of the VIP scores of the cellular lipid classes (Table 2). All of them reached significant values (VIP > 1), except cholesterol and monoacylglycerides contents, and contributed to the specificity of cell cultures incubated with MF from LCM and JM.

Consequently, we made the direct comparison of the lipid profiles of the MF with those of the cell cultures that were incubated with them (Figure 4). In this comparison set, cholesteryl esters increased in any of the cell cultures after incubation with MF, while free cholesterol decreased compared to the content in the micelles. It is well known that micellar cholesterol stimulates the activity of the acyl-coenzyme A:chlolesterol acyltransferase enzymes (ACAT) in a dose-dependent manner, as well as that only a minor fraction of the cholesterol entering the cell is esterified [36]. This fact is independent of the TAG and protein synthesis. The cell culture incubated with micelles from digested LCM showed a significantly lower cholesteryl ester content than the others, with cholesteryl ester content in the cell culture incubated with CM showing the highest value (Figure S1). We also observed that the free cholesterol content in the cell culture was significantly lower than that observed in the micelles. That is, micellar cholesterol might trigger the synthesis of the ACAT enzymes present on the cell culture after the exposition to micelles from digested LCM but, in the experimental conditions applied in this study, it was not enough to increase the cholesterol pool in the cells. On the other hand, the repeated increase in sphingomyelin, phosphatidylethanolamine, or phosphatidylcholine content in cell cultures compared to micelles denoted the second common response. These lipid classes are distinct indicators of the ubiquitous presence of BM-MFGM across all formulations and, consequently, digested micelles. In fact, the resistance to the digestion process of certain structures present in MFGM, such as the lipid rafts (rigid structures composed by sphingomyelin, cholesterol, and phospholipids) or ceramide, are considered as the explanation of the health benefits derived from the MFGM exert in the gut after the digestion [37,38,39]. Moreover, the increased presence of these polar lipids content in the differentiated Caco-2 cells presents relevant importance, as they form part of the “17 lipid signatures” described by Lefèvre-Arbogast [40] and their early dysregulation might contribute to cognitive decline diseases. In addition, the study describes that the supplementation of MFGM in food products counteracts the cognition and physical age-associated decline due to the presence of this polar lipid component in the MFGM. Moreover, their presence in the cell culture correlates with the described fusion process between the MFGM and the membrane of Caco-2 cells [41]. Therefore, there is clear evidence that the supplementation of BM-MFGM affects the metabolism of the cell, results that are in agreement with those of other studies that examined the effect of MFGM in murine models or other organisms with a higher complexity [42]. The third common pattern in any of the cell cultures was the increase in the cellular TAG content, in comparison with the micellar TAG content. This point was anticipated because the MFs contain the lipid units necessary for TAG synthesis, arising from BM-MFGM added to all the matrices [43], and from the sunflower oil-enrichment in the case of the LCM, but, indeed, two of the MFs were produced from digestion matrices containing carbohydrates, and processes inherent to the cell metabolism such as intestinal glycolysis, which also initiates TAG synthesis. The samples corresponding to the cell culture incubated with micelles from LCM showed the highest values in the TAG content (%) (Figure S2), with the cell culture incubated with MF obtained from digested JM raising the lowest values. On the other hand, the lipid class corresponding to the neutral lipids showed a significant decrease in the paired comparisons of lipids in MF and cell cultures for LCM and CM. However, the view of neutral lipids data in the cell cultures showed a wide range of data, with the highest values in the case of cell cultures incubated with MF isolated from digested CM, while cell cultures incubated with MF obtained from digested JM or LCM showed similar values for their neutral lipids content (Figure S3).

Therefore, comparisons of MF and cellular lipid profiles neither allowed the observation of the changes in the cellular lipid profiles nor represent data comparisons between cell culture groups, that is, it is necessary to perform data analysis of cell culture lipid profiles to clearly address the modulation of cellular lipid metabolism. There was a differential behavior of the LCM and both the JM and CM regarding cholesteryl esters, which significantly decreased in the former matrix but increased in the latter ones, while the TAG and neutral lipids content showed a homogenous performance, decreasing and increasing, respectively, in all the cell culture groups. This behavior was observed for the lipids profiles of cell cultures incubated with the supplemented food matrices in comparison with the control cells and with the cells incubated with non-supplemented food matrices. This divergence in so closely lipid features is due to the behavior of the free fatty acids plus cholesterol content that increased significantly in all the cell culture groups compared to the control cells. Additionally, among the lipid features that characterized the BM-MFGM, it is worth noting that phosphatidylcholine increased its presence in the treated cell cultures with micelles isolated from digested JM and CM, but not in the cells treated with the micellar fraction isolated from digested LCM, while polar lipids increased exclusively in the treated cell cultures with micelles isolated with CM (although this result was only observed in the comparison of supplemented cells with control ones). These results point to a diverging scenario regarding the lipid metabolism in the cell cultures. The implications of these findings are significant for the food industry, particularly in the formulation of functional foods that optimize the delivery of bioactive components like BM-MFGM. The interaction between food matrices and bioactive lipids must be carefully considered to ensure that the beneficial properties of these components are not masked.

However, while this study provides valuable insights, there are limitations that should be acknowledged. The in vitro nature of the study, while providing controlled conditions to study specific interactions, may not fully replicate the complex environment of the human gastrointestinal tract. Future studies should consider investigating the interaction between BM-MFGM and other food components, such as fiber or antioxidants, providing a more comprehensive understanding of how to optimize functional food formulations for health benefits.

## 5. Conclusions

The potential of in vitro digestion protocols in providing the food industry and academia with data for the identification of the suitable food matrix for supplementation with bioactive ingredients is fully exploited in this study emphasizing the influence of the food matrix on the bioaccessibility and the cellular assimilation of BM-MFGM. In this study, LCM matrices promoted the micellarization of TAG and neutral lipids, while polar lipids were predominantly found in CM and JM matrices, aligning with the lipid composition of BM-MFGM. These results highlight the importance for the food industry in identifying an optimal food matrix that preserves and does not mask the beneficial components of MFGM. Moreover, cholesteryl esters, sphingomyelin, phosphatidylethanolamine, and phosphatidylcholine seem to be the key lipid features that drive the adaptative process in the lipidomic adaptation of Caco-2 to the different digested food matrices. Consequently, implementation of cell culture models as a subsequent step to use the digested materials (mixed micelles) from the in vitro digestion protocol allows the identification of lipid descriptors that significantly feature the changes in cell metabolism, drawing potential in vivo adaptation of the intestinal epithelium to stimuli from digested food. Future research should aim to bridge the gap between in vitro and in vivo studies, further elucidating the mechanisms underlying these interactions and their implications for human health.

## Figures and Tables

**Table 1:** Compositional table of ingredients of each food matrix with the different levels of supplementation of BM-MFGM.

Ingredients	JM	CM	LCM
Jelly	3 g	-	-
All-purpose flour	-	150 g	150 g
Sunflower oil	-	-	80 g
Sugar	35 g	100 g	100 g
Water	62 g	-	-
Egg white	-	140 g	140 g
BM-MFGM	0, 2, 5, 10%	0, 2, 5, 10%	0, 2, 5, 10%

**Table 2:** Variable importance in projection scores (VIP) for lipid profiles in PLS-DA analysis for food matrices, micelles class isolated after digestion, and cell class treated with the corresponding micelles. Scores plots and correlation loadings are represented in Figure 1, Figure 2 and Figure 3. Performance of the models is detailed in Table S5. VIP scores correspond to first component of each model.

	Food Matrices Supplemented with BM-MFGM	Micelles Class	Cell Class
Lipid classes			
Triacylglycerides	2.610	-	1.706
Diacylglycerides	- ^2^	-	1.355
Free fatty acids plus cholesterol	-	1.187	1.544
Monoacylglycerides	-	1.623	-
Phosphatidylcholine	-	-	1.036
Total polar lipids ^1^	-	-	1.379
ƩPolar lipids	-	-	1.380
ƩNeutral lipids	2.678	2.105	2.633
Fatty acids			
C16:0	-	-	1.014
C18:1 c9	1.482	1.271	1.246
C18:2	1.990	1.826	1.916
ƩSaturated fatty acids	-	-	1.420
ƩMonounsaturated fatty acids	1.510	1.283	1.596
ƩPolyunsaturated fatty acids	2.003	1.779	1.968
ω6 fatty acids	1.983	1.779	1.916
ƩLong chain fatty acids	2.682	2.112	2.624
Triacylglycerides species			
CN50	-	-	1.418
CN52	1.287	-	2.124
CN54	2.304	-	2.086
ƩNeutral species	2.675	-	1.824
ƩPolar species	-	2.097	2.349
Cholesterol	-	1.597	-

^1^ Total polar lipids are the sum of individual phospholipids and sphingolipids content. ^2^ VIP scores < 1 for PLS-DA.

## Data Availability

Data will be available upon request.

## Supplementary Materials

The following supporting information can be downloaded at: https://www.mdpi.com/article/10.3390/nu16162798/s1, Figure S1. Relative amount of cholesteryl esters in cells treated with micellar fractions isolated from digested lipid and carbohydrate matrix (LCM), protein matrix (JM), and carbohydrate matrix (CM). Significant differences between pairs of data are marked (*p* < 0.05); Figure S2. Relative amount of triacylglycerides in cells treated with micellar fractions isolated from digested lipid and carbohydrate matrix (LCM), protein matrix (JM), and carbohydrate matrix (CM). Significant differences between pairs of data are marked (*p* < 0.05); Figure S3. Relative amount of neutral lipids in cells treated with micellar fractions isolated from digested lipid and carbohydrate matrix (LCM), protein matrix (JM), and carbohydrate matrix (CM). Significant differences between pairs of data are marked (*p* < 0.05). Table S1. Lipid composition of BM-MFGM; Table S2. Triacylglyceride species and cholesterol analyzed in the study, following the methodology described in [22], showing the corresponding numbers in Figure 1, Figure 2, Figure 3, Figure 4 and Figure 5; Table S3. Fatty acids analyzed in the study, following the methodology described in [23], showing the corresponding numbers in Figure 1, Figure 2, Figure 3, Figure 4 and Figure 5; Table S4. Lipid classes analyzed in the study, following the methodology described in [15], showing the corresponding numbers in Figure 1, Figure 2, Figure 3, Figure 4 and Figure 5. Table S5. PLS-DA performance considering lipid profile in BM-MFGM supplemented food matrices, in micelles from digested MFGM-supplemented food matrices or in cell cultures after incubation with those micellar fractions.
