# Supplementary material for: The Role of Food Matrices Supplemented with Milk Fat Globule Membrane in the Bioaccessibility of Lipid Components and Adaptation of Cellular Lipid Metabolism of Caco-2 Cells
Source: Nutrients. 2024 Aug 22;16(16):2798. doi: 10.3390/nu16162798 (PMC11357557; doi:10.3390/nu16162798)
Supplement: Supplementary file 1 [file nutrients-16-02798-s001.zip › Supplementary material.pdf]

Supplementary material of the manuscript entitled **The role of food matrices supplemented with milk fat globule membrane in the bioaccessibility of lipid components and adaptation of cellular lipid metabolism of Caco-2 cells**

Victoria Martínez-Sánchez[1], M. Visitación Calvo[2], J. Fontecha[2], Antonio Pérez-Gálvez[1]*

[1]Group of Chemistry and Biochemistry of Pigments, Instituto de la Grasa (CSIC), Building 46, 41013, Sevilla, Spain.

[2]Food Lipid Biomarkers and Health Group, Institute of Food Science Research (CSIC-UAM), 28049, Madrid, Spain.

*Author for correspondence at aperez@ig.csic.es. Phone: +34954611550.

Figure S1. Relative amount of cholesteryl esters in cells treated with micellar fractions isolated from digested lipid- and carbohydrate matrix (LCM), protein matrix (JM) and carbohydrate matrix (CM). Significant differences between pairs of data are marked ($P<0.05$).

Figure S2. Relative amount of triacylglycerides in cells treated with micellar fractions isolated from digested lipid- and carbohydrate matrix (LCM), protein matrix (JM) and carbohydrate matrix (CM). Significant differences between pairs of data are marked ($P<0.05$).

Figure S3. Relative amount of neutral lipids in cells treated with micellar fractions isolated from digested lipid- and carbohydrate matrix (LCM), protein matrix (JM) and carbohydrate matrix (CM). Significant differences between pairs of data are marked ($P<0.05$).

Table S1. Lipid composition of BM-MFGM.

Total lipids (%)	11.61 ± 0.97
Lipid classes (% of total Lipids)	
Cholesteryl esters	-
Triacylglycerides	65.84 ± 0.90
Diacylglycerides	16.77 ± 0.68
Free fatty acids *plus* cholesterol	2.35 ± 0.14
Monoacylglycerides	0.16 ± 0.02
Glucosylceramides	-
Lactosylceramides	0.03 ± 0.01
ΣPolar lipids	14.88 ± 0.31
Phospholipids (% of total Polar lipids)	
Phosphatidylethanolamine	34.02 ± 0.21
Phosphatidylinositol	3.82 ± 0.26
Phosphatidylserine	17.55 ± 0.16
Phosphatidylcholine	30.94 ± 0.20
Sphingomyelin	13.67 ± 0.16
Fatty Acids (% of Total Lipids)	
ΣSaturated fatty acids	65.06 ± 0.40
ΣMonounsaturated fatty acids	30.23 ± 0.31
ΣPolyunsaturated fatty acids	4.71 ± 0.24
C4:0	2.37 ± 0.18
C6:0	1.67 ± 0.13
C8:0	0.92 ± 0.09
C10:0	1.98 ± 0.19
C10:1	0.13 ± 0.02
C12:0	2.51 ± 0.18
C14:0	9.30 ± 0.34
C14:1	0.50 ± 0.04
C15:0 *anteiso*	0.10 ± 0.01
C15:0 *iso*	0.26 ± 0.01
C15:0	0.85 ± 0.02
C16:0 *iso*	0.13 ± 0.01
C16:1 *c9*	1.43 ± 0.04
C16:0	31.91 ± 0.44
C17:0 *anteiso*	0.21 ± 0.01
C17:0	0.41 ± 0.03
C17:1	0.07 ± 0.01
C18:0	12.36 ± 0.48
ΣC18:1 *trans*	1.29 ± 0.11
C18:1 *c9*	25.94 ± 0.31
C18:1 *c11*	0.88 ± 0.09
C18:2 *trans-trans*	0.14 ± 0.02
C18:2 (LA, ω6)	0.25 ± 0.04
C18:3 (ALA, ω3)	0.16 ± 0.01
CLA	0.14 ± 0.01
C20:0	0.22 ± 0.02
C20:3 ω6 (DGLA)	0.03 ± 0.01
C20:4 ω6 (AA)	0.14 ± 0.01
C20:5 ω3 (EPA)	0.03 ± 0.01
C22:5 ω3 (DPA)	0.06 ± 0.01

Table S2. Triacylglyceride species and cholesterol analysed in the study, following the methodology described in [22], showing the corresponding numbers in Figures 1-5.

Lipid compound	Number in Figures 1-5
CN24	1
CN26	2
CN28	3
CN30	4
CN32	5
CN34	6
CN36	7
CN38	8
CN40	9
CN42	10
CN44	11
CN46	12
CN48	13
CN50	14
CN52	15
CN54	16
ΣNeutral species	17
ΣPolar species	18
Cholesterol	19

Table S3. Fatty acids analyzed in the study, following the methodology described in [23], showing the corresponding numbers in Figures 1-5.

Fatty acid	Number in Figures 1-5
C10:0	20
C10:1	21
C12:0	22
C14:0	23
C15:0 ai	24
C14:1 *c9*	25
C16:0 ai	26
C16:0	27
C17:0 i	28
C16:1 *c9*	29
C17:0 ai	30
C18:0i	31
C17:1 c10	32
C18:0	33
C18:1 *c9*	34
C18:1 *c11*	35
C19:0	36
C18:2	37
C18:3	38
C18:4 ω3	39
C20:1	40
C22:0	41
C20:3 ω6 (DGLA)	42
C20:4 ω6 (AA)	43
C20:5 ω3 (EPA)	44
C22:5 ω3 (DPA)	45
C24:0	46
C22:6 ω3 (DHA)	47
ΣSaturated fatty acids	48
ΣMonounsaturated fatty acids	49
ΣPolyunsaturated fatty acids	50
ω3 fatty acids	51
ω6 fatty acids	52
ΣShort chain fatty acids	53
ΣMedium chain fatty acids	54
ΣLong chain fatty acids	55

Table S4. Lipid classes analyzed in the study following the methodology described in [15], showing the corresponding numbers in Figures 1-5.

Lipid class	Number in Figures 1-5
Cholesteryl esters	56
Triacylglycerides	57
Diacylglycerides	58
Free fatty acids *plus* cholesterol	59
Monoacylglycerides	60
Glucosylceramides	61
Gangliosides	62
Lactosylceramides	63
Phosphatidic acid	64
Phosphatidylethanolamine	65
Phosphatidylinositol	66
Phosphatidylserine	67
Phosphatidylcholine	68
Sphingomyelin	69
Lyso-Phospholipids	70
ΣPolar lipids	71
Total polar lipids[1]	72
ΣCeramides	73
ΣNeutral lipids	74

[1]Lipid classes content includes, the total polar lipids (ΣPolar lipids plus lyso-phospholipids), the ceramides and the neutral lipids.

Table S5. PLS-DA performance considering lipid profile in BM-MFGM supplemented food matrices, in micelles from digested MFGM-supplemented food matrices or in cell cultures after incubation with those micellar fractions.

	**Accuracy**[a]	**R^2**	**Q^2**
Lipids in supplemented food matrices	1.0	0.942	0.901
Lipids in micelles	0.883	0.879	0.840
Lipids in cells	1.0	0.912	0.883

[a]Calculated for 2 components.
