# Supplementary figures and images for: The Role of Food Matrices Supplemented with Milk Fat Globule Membrane in the Bioaccessibility of Lipid Components and Adaptation of Cellular Lipid Metabolism of Caco-2 Cells
Source: Nutrients. 2024 Aug 22;16(16):2798. doi: 10.3390/nu16162798 (PMC11357557; doi:10.3390/nu16162798)

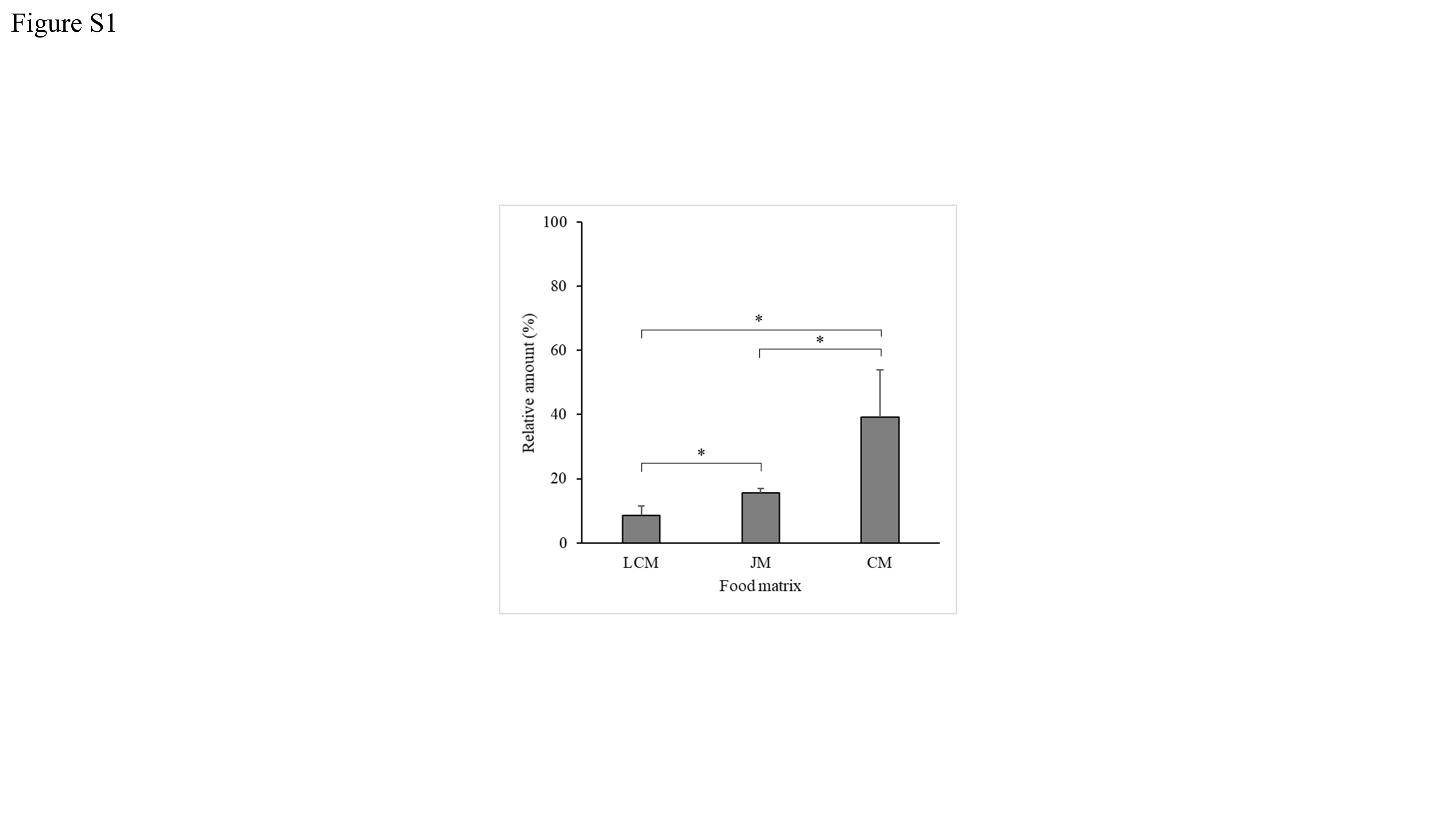

Supplement: Supplementary file 1 [file nutrients-16-02798-s001.zip › FigureS1.jpg]

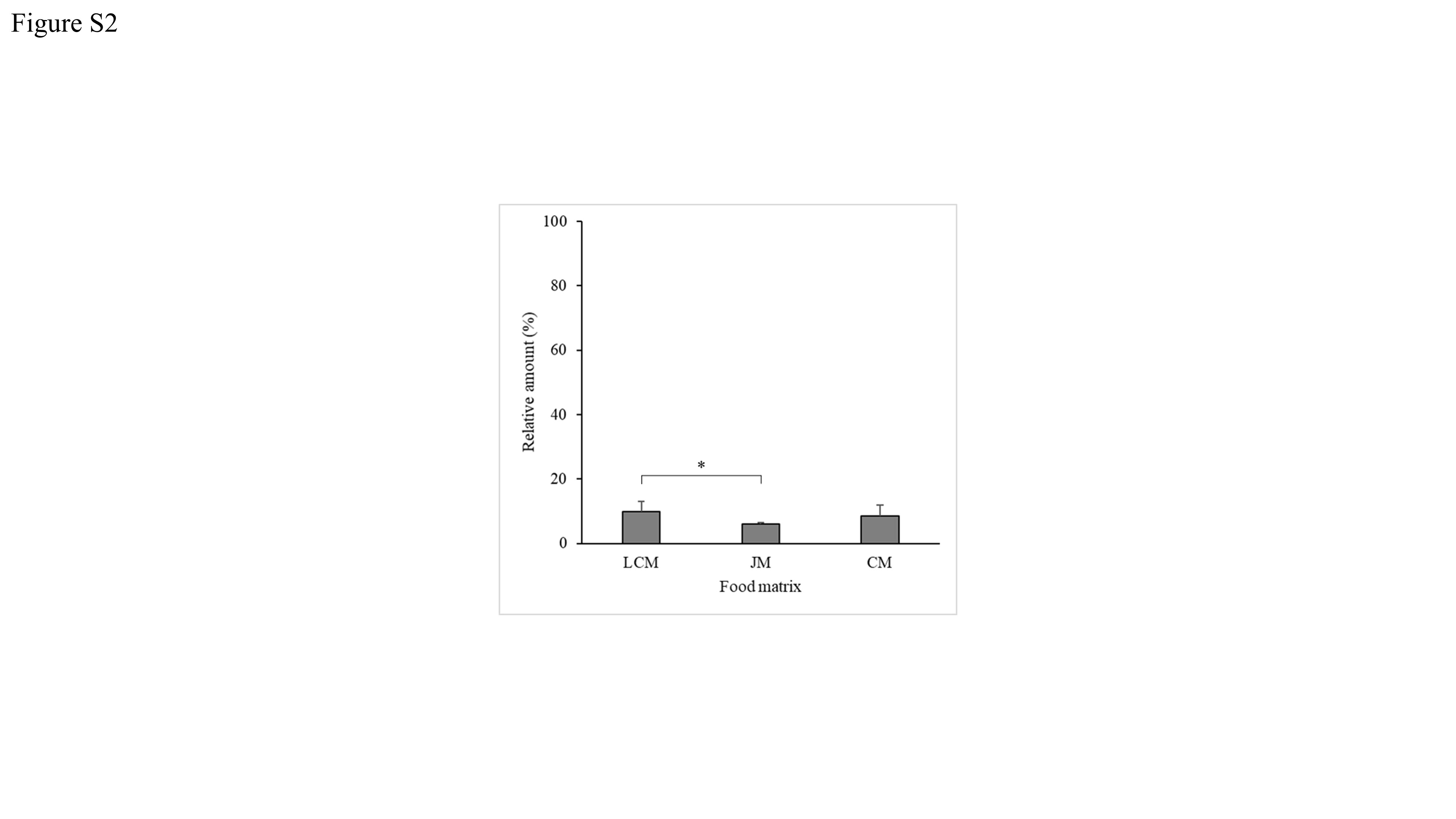

Supplement: Supplementary file 1 [file nutrients-16-02798-s001.zip › FigureS2.jpg]

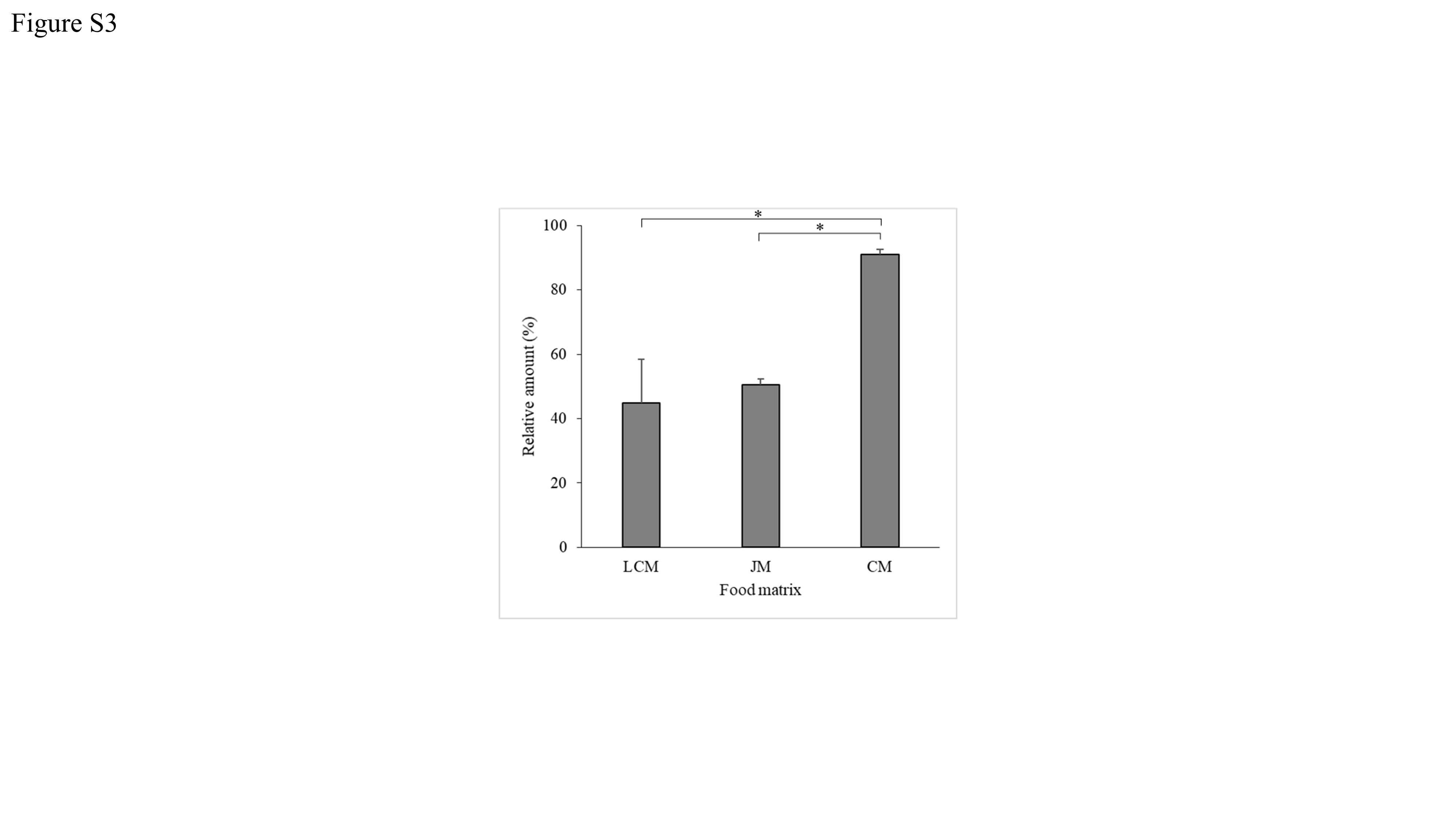

Supplement: Supplementary file 1 [file nutrients-16-02798-s001.zip › FigureS3.jpg]
